# Genetic Alteration Profiling in North Macedonian Lung Cancer Patients

**DOI:** 10.3390/genes16101177

**Published:** 2025-10-10

**Authors:** Aleksandar Eftimov, Rubens Jovanovic, Slavica Kostadinova Kunovska, Magdalena Bogdanovska Todorovska, Boro Ilievski, Panche Zdravkovski, Selim Komina, Blagica Krstevska, Simonida Crvenkova, Marija Simonovska, Gordana Petrushevska

**Affiliations:** 1 Institute of Pathology, Faculty of Medicine, Ss. Cyril and Methodius University, 1000 Skopje, North Macedonia; 2 University Clinic of Radiotherapy and Oncology, Medical Faculty, 1000 Skopje, North Macedonia

**Keywords:** lung cancer, *BRAF*, *KRAS*, *EGFR*, gene alterations

## Abstract

**Background/Objectives**: Late diagnosis and inefficient treatment regimens lead to poor prognosis, with a low 5-year survival rate for both non-small-cell lung cancer (NSCLC) and small-cell lung cancer (SCLC). New targeted therapeutic agents can be developed and introduced only by first discovering new driver oncogenes and with a thorough investigation of the known driver genes. The aim of the current study is to investigate the prevalence of alterations in the eight most frequently altered genes in lung cancer—*BRAF*, *EGFR*, *KRAS*, *ALK*, *ROS1*, *HER2*, *PD-L1* and *PIK3CA*. **Methods**: Real-time polymerase chain reaction (RT-PCR) was used to detect *KRAS* and *EGFR* mutations, multiplex PCR and microarray hybridization for *KRAS*/*BRAF*/*PIK3CA* mutations. Immunohistochemical analysis was performed for the detection of *ALK*, *HER2/NEU*, *ROS-1* and *PD-L1* alterations. **Results**: Overall, 221/603 patients (36.65%) had at least one genetic alteration, of which 22 patients (3.65%) had two genetic alterations and two patients had more than two genetic alterations. Additionally, 50 patients were identified with one or more *KRAS* mutations (8.29%), 45 patients with *EGFR* mutations (7.46%), and 1.82% with *PIK3CA* mutations and 0.66% with *BRAF* mutations. Furthermore, 50% of the co-occurring alterations were either on *KRAS* and *PIK3CA* genes (3/6), on *KRAS* and *BRAF* genes (2/6, 33.33%) or on *EGFR* and *PIK3CA* genes (1/6, 16.67%), and 10.45% of the patients exhibited *PD-L1* overexpression, 5.31% *ALK* rearrangements, and 2.36% *HER2/NEU* expression, with no *ROS-1* rearrangements detected. **Conclusions**: Comprehensive testing for somatic alterations in *EGFR*, *BRAF*, *KRAS*, and *PIK3CA* is significant in guiding therapeutic decisions in lung cancer management. Such testing should be routinely conducted to establish a thorough genetic profile of lung cancers in a manner that is both time-efficient and cost-effective.

## 1. Introduction

The World Health Organization (WHO)’s International Agency for Research on Cancer (IARC) GLOBOCAN cancer statistics 2022 reports lung cancer as the leading cause of cancer death, with an estimated 1.8 million deaths (18%). It is the most frequently diagnosed cancer worldwide, with 2,480,675 new cases in 2022. The figures are sharply rising in the female population and in emerging countries [1]. In North Macedonia in 2022, lung cancer was both the most commonly diagnosed cancer and the leading cancer death cause, with 1150 new lung cancer cases, representing 15.2% of all new cancer cases diagnosed in 2022 and 1002 deaths [2].

Based on the National Comprehensive Cancer Network (NCCN) Guideline, 85–90% of lung cancers are caused by voluntary or involuntary (secondary) cigarette smoking [3]. Other possible risk factors include disease history (e.g., chronic obstructive pulmonary disease (COPD)), cancer history, family history of lung cancer, and exposure to other carcinogens [4,5], including nickel, silica, fumes, diesel, beryllium, arsenic, cadmium, chromium and asbestos [6,7]. The majority of patients either have metastatic disease at diagnosis or later experience a relapse [8]. In such patients, symptom control, quality of life and prolonged overall survival (OS) are achieved with palliative systemic therapies.

The histological characterization of lung cancer is the cornerstone for selecting systemic therapies. The vast majority of lung cancers (approximately 80%) are non-small-cell lung cancer (NSCLC), including two major types [9]: **(1)** non-squamous carcinoma, including large-cell lung carcinoma (LCLC), large-cell neuroendocrine carcinoma (LCNEC), adenocarcinoma of lungs and/or *bronchii*, with or without metastases (e.g., on supraclavicular gland, liver, abdominal, *ms ossei c. vertebrae*, pleurae, cerebral), and lung cancer not otherwise specified (NOS), and **(2)** squamous cell (epidermoid) carcinoma of the principal *bronchii* and/or lungs [10]. The remaining 20% are small-cell lung cancers (SCLCs). Lung cancer classifications are enhanced through the validation of recurring oncogenic mutations, which serve as predictive biomarkers for identifying treatable oncogenic dependencies. Understanding lung cancer morphology is essential due to its diverse spectrum, with many atypical characteristics that complicate accurate diagnosis. In this context, comprehensive genomic analyses are conducted to identify various predictive and prognostic biomarkers, as well as gene expression profiles. The resulting data facilitate the differentiation of cancer subgroups based on molecular phenotypes [11], which is vital for the selection of appropriate treatment strategies.

The primary treatment modalities for patients with NSCLC include surgery, radiotherapy (RT), and systemic therapy. These approaches may be utilized individually or in combination, contingent upon the specific status of the disease. Surgical resection is considered the standard care for patients with stage I or II disease and for single-station non-bulky IIIA disease (N2, metastasis in ipsilateral mediastinal and/or subcarinal lymph node(s)) [12]. For IB (tumor ≥ 4 cm) to IIIA patients, it is usually followed by platinum-doublet chemotherapy, with very few trials showing the efficacy of adjuvant immunotherapy in stage IB to IIIA and several additional ongoing trials evaluating adjuvant oral tyrosine kinase inhibitors (TKIs) or immunotherapy [12]. RT, preferably stereotactic ablative radiotherapy, is recommended for I-IV stage patients in different regimes; the doses and duration depend on the NSCLC stage (e.g., in locally advanced NSCLC; in early-stage NSCLC with contraindications for surgery, e.g., major medical comorbidity, severely limited lung function; or as preoperative or postoperative therapy, as therapy for limited recurrences and oligometastases or as palliative therapy for patients with incurable NSCLC) [13,14,15,16]. The standard systemic therapy for NSCLC typically involves platinum-doublet chemotherapy. This regimen includes the use of cisplatin or carboplatin in combination with pemetrexed for patients with non-squamous metastatic NSCLC. For patients with squamous metastatic NSCLC, cisplatin or carboplatin may be administered alongside either paclitaxel or gemcitabine [12]. The platinum-based combinations have comparable efficacy and low overall response rate (RR), ranging between 17 and 32% [17]. The results are less than satisfactory, as sustainable disease control is infrequently attained due to either intrinsic or acquired drug resistance. For such patients, concurrent or sequential target immunotherapy is crucial. Nonetheless, it has been observed that approximately 85–90% of patients with advanced NSCLC do not exhibit oncogenic alterations that are amenable to targeted therapies, which is associated with a poorer prognosis and an elevated incidence of local relapse [18]. For them, treatment options include chemotherapy or single or combined immunotherapy [12]. Immunotherapeutic agents used in NSCLC are the immune checkpoint inhibitors (ICIs); three *PD-1* inhibitors, cemiplimab-rwic, nivolumab and pembrolizumab; and one programmed (cell) death-ligand 1 (*PD-L1*) antibody, atezolizumab [3]. The survival rates and duration of response tend to improve with higher levels of *PD-L1* expression [12].

Currently, there is no established role for additional systemic therapy following adjuvant chemotherapy. However, recent data concerning further adjuvant treatment with immunotherapy and tyrosine kinase inhibitors (TKIs) have raised important questions regarding the existing standard of care [12]. Several molecular alterations, defined as driver mutations, have been targeted with >20 targeted agents approved for the treatment of advanced NSCLC. Such are the alterations in *BRAF*, epidermal growth factor receptor (*EGFR*) gene, anaplastic lymphoma kinase (*ALK*) gene, Kirsten Rat Sarcoma virus (*KRAS*), repressor of silencing 1 (*ROS-1*) and *PD-L1* for squamous cell carcinoma. Mutations in phosphatidylinositol-4,5-bisphosphate 3-kinase catalytic subunit alpha (*PIK3CA*) and human epidermal growth factor receptor 2 (*HER2*) have lower frequency, but have emerging therapeutic relevance. Furthermore, the identified key mechanisms of drug resistance in NSCLC indicate that additional mutations in tumor-driving genes may contribute to drug insensitivity and facilitate tumor progression following treatment [18]. Typically, mutations and alterations are observed in a non-overlapping manner; however, between 1% and 3% of NSCLC cases may exhibit concurrent alterations [3].

Guidelines for NSCLC advocate for the assessment of driver mutations in all non-squamous tumors. It is important to note that driver mutations are often considered in individuals without a significant smoking history, as well as in those with a minimal smoking history. Furthermore, tumors of squamous histology in non-smokers should be evaluated for testing on an individual basis [12]. The International Association for the Study of Lung Cancer recommends, at a minimum, testing for *EGFR*, *ALK*, and *ROS1*. Recent guidelines suggest the inclusion of additional tests for *BRAF*, *KRAS*, *MET*, *ERBB2*, and *RET* [3,19]. It is also advised that *ROS1* testing be conducted for all patients diagnosed with *adenocarcinoma*, and *BRAF* testing should be considered for patients with metastatic non-squamous NSCLC or NSCLC NOS [3].

The aim of this non-randomized study is to investigated the gene expression and mutation signatures of different subtypes of lung cancers in 603 North Macedonian patients, including NSCLC (adenocarcinoma, squamous-cell carcinoma) and SCLC. Patients were examined for presence of genetic alterations in ≥2 of the following genes: *EGFR*, *BRAF*, *KRAS*, *ALK*, *ROS1*, *HER2*, *PD-L1* and *PIK3CA*.

## 2. Materials and Methods

### 2.1. Sample Collection

This non-randomized, open-trial study included 603 patients with different types of lung cancers, treated at the University Clinic of Oncology and Radiology, UCC Skopje, from 17 November 2020 to 23 February 2025. All biopsies are performed at an early treatment stage. Tumor histology and stage were estimated according to WHO and pTNM classification according to the Union of International Cancer Control (UICC) [20]. All included patients had tissue samples taken at the Institute of Pathology at the Faculty of Medicine, University Ss. Cyril and Methodius, Skopje, North Macedonia. All patients gave their written informed consent to participate in the study. The Ethics Committee for research involving people at the Faculty of Medicine, UKIM, in Skopje, North Macedonia, approved investigation with human subjects according to The Code of Ethics of the World Medical Association—Declaration of Helsinki.

### 2.2. Demographic Properties of the Patients Included in the Study

The age of the lung cancer patients enrolled ranged from 14 to 86 years (mean 62.56 ± 9.34), of which 70.31% (*n* = 424) were male and 29.68% (*n* = 179) were female (Table 1). According to the histology, 99.17% (*n* = 598) patients had NSCLC, and only 0.83% (*n* = 5) had SCLC. Of the 598 NSCLC patients, 560 (92.87%) had been diagnosed with adenocarcinoma, 27 (4.47%) had been diagnosed with squamous-cell carcinoma and 11 (1.82%) had been diagnosed with LCC. Metastatic NSCLC was diagnosed in 31 patients, 13 of whom had metastases in lymph nodes (mediastinal, supraclavicular) alone, or together with hepatic or pulmonary metastases; brain metastases was present in 4 patients, liver and pleural metastases were present in 3 patients each, 2 patients had either metastases in mediastinum or bone, and 1 patient had abdominal metastasis, 1 *pul.bill* and bone metastasis and 1 *pul.bill* and liver metastasis. Additional comprehensive details regarding the patients are presented in Table 1.

### 2.3. Molecular Testing

Extraction of DNA from formalin-fixed paraffin-embedded (FFPE) samples was performed using Cobas^®^ DNA Sample Preparation Kit (Roche Diagnostics, Basel, Switzerland). The DNA concentration was determined using the ScanDrop2 instrument (Analytik Jenna, Jena, Germany) spectrophotometer and the dilution of isolated DNA was carried out in accordance with the protocol for mutation detection. All samples had appropriate DNA quality/yield.

Detection of *EGFR* mutations in exons 18–21 (exon 18: G719X (G719A, G719C, and G719S); exon 19: deletions and complex mutations; exon 20: S768I, T790M, and insertions; and exon 21: L858R and L861Q) was performed using Cobas^®^ *EGFR* Mutation Test V2 (Roche Diagnostics, Basel, Switzerland) on Cobas Z480 IVD real time-polymerase chain reaction (RT-PCR) (Roche Diagnostics, Basel, Switzerland). In order to detect mutations, 2 ng/L isolated DNA was used according to the manufacturer’s protocol. A mutant control and negative control were included in each run to confirm its validity. In FFPE tissue analysis, the Cobas^®^ EGFR Test can detect mutations with a 5% mutation level using a standard input of 50 ng per reaction well.

Mutations in *KRAS*, *BRAF* and *PIK3CA* genes were detected using *KRAS*/*BRAF*/*PIK3CA* Array (Randox Bioscience, Crumlin, UK), using Randox Evidence Investigator System (Randox Bioscience, Crumlin, UK). The examination relies on a combination of multiplex PCR and microarray hybridization for the detection of *BRAF* V600E point mutation, 20-point mutations in codons 12, 13, 61, and 146 of the *KRAS* gene, including known resistance mutations (G12A, G12R, G12D, G12C, G12S, G12V, G13D, G13C, G13R, Q61K, Q61L, Q61R, Q61H1, Q61H2, A146T, A146P), and three-point mutations in the *PIK3CA* gene (E542K, E545K, H1047R). For some samples, the detection of a V600E (1799T>A) mutation of the *BRAF* gene was performed using a Cobas^®^ 4800 *BRAF* V600E Mutation IVD Test (Roche Diagnostics, Basel, Switzerland) on Cobas Z480 IVD RT-PCR (Roche Diagnostics, Bssel, Switzerland), with 96.4–99.3% sensitivity and 80.0–99.4% specificity.

### 2.4. Immunohistochemical (ICH) Analysis

Immunohistochemical staining was conducted following the protocol provided by Ventana Medical Systems (Oro Valley, AR, USA) and Agilent Technologies (Santa Clara, CA, USA). The detection of *ALK* protein expression via ICH is executed using the primary antibodies against ALK (rabbit, monoclonal, clone D5F3; Cat. No. 06785042001, Ventana Medical Systems, AR, USA), utilizing the Ventana Benchmark ULTRA automated stainer (Ventana Medical Systems, AR, USA). The immunohistochemical detection of *PD-L1* protein expression is carried out using the *PD-L1* IHC 22C3 pharmDx Kit (Agilent Technologies, CA, USA) along with the EnVision FLEX visualization system on the Autostainer Link 48 (Agilent Technologies, CA, USA). The detection of *HER-2* protein expression through immunohistochemistry was performed using the antibodies against HER-2/NEU (rabbit, monoclonal; clone 4B5; Cat. No. 05278368001, Ventana Medical Systems, AR, USA), on the Ventana BenchMark ULTRA automated stainer (Ventana Medical Systems, AR, USA). It is essential to exclude known staining elements, which include light cytoplasmic stippling in alveolar macrophages, cells of neural origin (nerve and ganglion cells), glandular epithelial staining, and cells within lymphocytic infiltrate. Additionally, some background staining may be noted within normal mucosa in NSCLC (including mucin) and in necrotic tumor regions, which were also excluded from the clinical assessment.

### 2.5. Statistical Methods

Descriptive statistics were determined to summarize the mutation frequencies of the genes, along with calculations of the mean age of patients per each specific mutation.

## 3. Results

**Figure 1:**
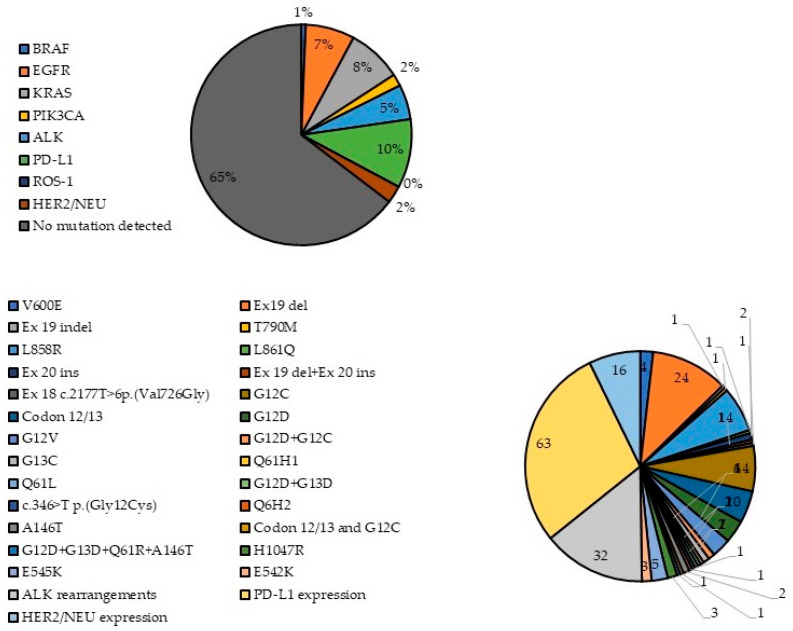
Frequency of detected gene mutations.

The overall results from the genetic profiling of lung cancer patients are given in Table 2 and Figure 1. Overall, 221/603 patients (36.65%) had at least one genetic alteration; 432 patients were tested for the presence of the *BRAF V600E* mutation, and it was only found in 4 patients (0.93% of tested). Three of them were male and one female; three had *adenocarcinoma* and one *squamous cell carcinoma*. Further, 527 patients were tested for *EGFR* mutations and 45 (8.54%) tested positive, 22 of whom (48.89%) were women and 23 (51.11%) men, with average age of 61.36 years. All 45 patients were diagnosed with *adenocarcinoma.* More than half of them (*n* = 24, 53.33%) had exon 19 deletion, and 14 patients (31.11%) had the *L858R* mutation on exon 21. Exon 20 insertions were detected in two patients, while each of the following mutations was observed in only one patient: exon 19 indel, *T790M*, *L816Q*, exon 19 deletion + exon 20 insertion and exon 18 c.2177T>p. (Val726Gly). In addition, 193 patients were tested for *KRAS* mutations. Of them, 50 (25.91%) had at least one *KRAS* mutation; 34 (68.00%) were male and 16 (32.00%) were female, with average age of 61.25 years. The majority of patients were diagnosed with *adenocarcinoma* (*n* = 44, 88%), two with squamous-cell *carcinoma* and one with *adenosquamous carcinoma* and LCLC. The detected mutations included G12C (28%), codon 12/13 mutation (20%), G12D (14%), G12V (12%), G13C (4%), both G12D and G12C (4%), A146T (4%) and Q61H1, Q61L, Q6H2, c.346>T p. (Gly12Cys), and the co-appearance of G12D and G13D, codon 12/13 and G12C and G12D, G13D, Q61R and A146T, each detected in one patient (2%). Furthermore, 142 patients were tested for *PIK3CA* mutations. Of them, mutations were present only in 11 (7.75%) of the patients; 6 men and 5 women, with an average age of 66.91 years. Nine of the patients were diagnosed with adenocarcinoma, one with planocellular cancer and one with squamous cell cancer. Six of the patients (54.55%) had E545K mutation, E542K was present in three patients (27.27%), and H1047R was present in two of them (18.18%).

Also, 411 patients were tested for *ALK* rearrangements; of them, 32 (7.79%) patients tested positive, 22 male and 10 female, with an overall average age 60.94 years. Twenty-six of them had adenocarcinoma, three planocellular cancer, three squamous cell cancer and one patient had LCLC. Additionally, 264 patients were tested for *PD-L1* expression; 63 (23.86%) had *PD-L1* expressed, and 48 (76.19%) were male and 15 (23.81%) female, with an average age of 63.12 years. SCLC, *adenosquamous carcinoma* and LCLC were present in one patient each, four patients had squamous cell carcinoma, six had planocellular *carcinoma*, while most of the patients (50, 79.37%) were diagnosed with *adenocarcinoma*. Moreover, 117 patients were tested for the presence of *ROS-1* rearrangements, but none of them had one. Finally, 144 patients were examined for *HER2* rearrangements, which were present in 16 patients (11.11%), 12 of whom were men and 4 women, with an overall mean age of 63.81 years. Nine patients had adenocarcinoma, four squamous cell *carcinoma*, one patient had planocellular cancer and one had LCLC.

Of the 603 patients, concomitant alterations of up to three different genes were observed in 22 patients (3.65%, n = 603) (Table 3). All patients were diagnosed with adenocarcinoma, 16 men and 7 women with an average age of 63.04 years. *PD-L1* rearrangement is the most frequent mutual alteration, and was present in half of the tested patients, and in five cases with *ALK* rearrangements, which were also frequent (36.36% of the patients with concomitant alterations). In the other four cases, they co-occurred with *KRAS* mutations (G12V, codon 12/13), and in one case each with *PIK3CA* (E545K), *BRAF* (V600E), *HER2* and *EGFR* mutations. Other co-occurring alterations included two patients with *KRAS/PIK3CA* (G12C, G12D/H1047R and G12C/E542K) and two patients with *KRAS* (G12C or c.346G>T)/*ALK*, *KRAS* (G12C)/*HER2*, *KRAS* (G12C)/*BRAF*, *EGFR* (L858R)/*PIK3CA* (H1047R) and *ALK/PIK3CA* (E545K). Finally, two patients had co-occurrence of three mutations: *KRAS* codon 12/13, *ALK* and *PD-L1* rearrangements. All other details are given in Table 3.

## 4. Discussion

An individualized treatment approach to NSCLC starts with an accurate pathological diagnosis, imaging methods, endoscopic techniques for tissue sampling and staging according to pTNM classification, and the establishment of the correct tumor genotype. Thorough genomic analyses and functional preclinical validation are crucial for broadening the spectrum of potentially actionable genomic aberrations. According to current guidelines, NSCLC patients are screened for treatable oncogenic alterations for the purpose of proper treatment selection. The patients from our study were tested for the following gene expression alterations, in descending order: *EGFR* (87.07%), *BRAF* (71.52%), *KRAS* (31.95%) and *PIK3CA* (23.51%). Most of the patients had one or more *KRAS* mutations (8.28%, *n* = 603), followed by *EGFR* mutations (5.13%, n = 603), *PIK3CA* mutations (1.82%, n = 603) and *BRAF* mutations (0.66%, *n* = 603). It is anticipated that this proportion will increase as the scope of genotype-specific clinical trials expands at our institution and other centers.

### 4.1. EGFR Mutations

The identification of activating *EGFR* mutations, along with the associated increased sensitivity to *EGFR* TKIs, represents a critical initial step in the implementation of targeted treatment strategies for lung cancer. *EGFR* is a receptor tyrosine kinase that is typically located on the surface of epithelial cells and is frequently overexpressed in various human malignancies. The NCCN NSCLC Panel advocates for the testing of *EGFR* mutations and other biomarkers in patients diagnosed with metastatic non-squamous NSCLC or NSCLC NOS, as well as in cases of squamous cell carcinoma [21,22,23,24].

The most prevalent *EGFR* mutations consist of deletions in exon 19, which occur in 45% of patients with *EGFR* mutations, and the p.L858R point mutations in exon 21, found in 40% of patients with *EGFR* mutations. These mutations are linked to a favorable response to oral *EGFR* TKIs [25,26]. Both mutations are observed in 10% of Caucasian patients with NSCLC and up to 50% of Asian patients [27]. The majority of patients exhibiting common EGFR mutations are typically nonsmokers or former light smokers and have been diagnosed with adenocarcinoma histology [28], and are also of younger age and female sex [29], although some studies show no statistically significant association between sex, histologic subtype and racial differences and *EGFR* mutation status [30,31], or with age, performance status or smoking status [31]. While both mutations are considered comparable in their predictive value for the efficacy of *EGFR* TKIs, two studies indicate that patients with exon 19 deletions experience a more significant benefit from *EGFR* TKIs compared to those with exon 21 L858R substitutions. Specifically, the therapeutic effect is found to be 50% greater in the former group, accompanied by an extended overall survival rate [21,32], and response to platinum-based chemotherapy was observed only in patients with exon 19 mutations (RR = 46% versus 0%, *p* = 0.02) [33].

Other mutations in *EGFR* exon 20 comprise a diverse array of on-frame duplications and insertions, including insASV, insSVD, and insNPH. These mutations are observed in approximately 2% of patients with NSCLC and in 4–12% of patients with *EGFR* mutations [34,35,36,37]. These patients have variable low response rates (9–25%) to erlotinib, afatinib or gefitinib [34,38], depending on the specific *EGFR* exon 20 insertion mutation [34,39,40], although exceptions exist (e.g., p.A763_Y764insFQEA) [35,41,42]. First-line platinum-based chemotherapy, with or without immunotherapy, is also recommended for patients exhibiting EGFR exon 20 mutations, such as carboplatin in combination with either pemetrexed or paclitaxel [43,44]. In this study, the frequency of *EGFR* mutations was 7.46%; almost equally present in both sexes. Exon 19 deletions and L858R were the two most commonly observed alterations, with only one patient with a rare exon 20 mutation (L816Q), one patient with T790M and one with concomitant exon 19 and 20 *EGFR* mutation.

### 4.2. BRAF Mutations

*BRAF* is a serine/threonine kinase and is an integral component of the canonical *MAP/ERK* signaling pathway. Activating mutations in *BRAF* lead to dysregulated signaling within this pathway. The specific *BRAF* point mutation resulting in an amino acid change at position 600 (p. V600E) has been linked to a favorable response to combined therapeutic approaches utilizing oral inhibitors of *BRAF* and *MEK*, such as dabrafenib and trametinib. Additionally, other non-V600E mutations have been identified in NSCLC at a frequency comparable to that of V600E mutations [45]; however, the implications of these mutations for therapy selection remain unclear, and as a result, specific targeted therapies are not currently available [3]. Here, 603 of the patients were assessed only for V600E mutation, with a frequency of 0.66%. Testing for other, non-*BRAF V600E* mutations, however, should be carried out in larger cohort study in order to explore their impact on guiding personalized treatment regimens.

### 4.3. KRAS Mutations

*KRAS* is a G-protein characterized by its intrinsic GTPase activity; mutations that activate *KRAS* lead to unregulated signaling within the MAP/ERK pathway. Approximately 25% of the patients with adenocarcinoma in a North American population have *KRAS* mutations [25,30]. The prevalence of *KRAS* mutations is linked to cigarette smoking, in contrast to many other actionable mutations [46]. Substitutions at *KRAS* positions G12, G13, and Q61 decrease the enzyme’s ability to hydrolyze GTP, whereas the A146T mutation enhances the formation of *KRAS*-GTP by increasing nucleotide exchange. This alteration subsequently diminishes the oncogenic potential of this isoform [47]. The presence of a *KRAS* mutation, particularly at codon 12 (*KRAS* G12C), is associated with poor survival outcomes and reduced effectiveness of *EGFR* TKI therapy [25,30,48]. However, these mutations do not serve as reliable predictors of RR, progression-free survival (PFS), or overall survival in patients with lung cancer. Additionally, *KRAS* mutations exhibit lower sensitivity to chemotherapy compared to wild-type *KRAS* [47]. It is important to note that *KRAS* mutations typically do not coexist with genetic variants such as *EGFR*, *ROS1*, *BRAF*, and *ALK*. Consequently, *KRAS* testing can help identify patients who may not benefit from additional molecular biomarker assessments [3].

In this study, various *KRAS* mutations were detected in 50 patients (8.29%), approximately 90% of them on codon 12, which is in accordance with previously published data [47]. The most prevalent *KRAS* mutations identified were G12C and G12D, representing 20% and 14% of all *KRAS* mutations, respectively. Additional *KRAS* mutations included G12V (12%), Q61H (2%), Q61L (2%), Q6H2 (2%), G13C (2%), and c.346G>T (2%). When analyzing *KRAS* mutations with Cobas^®^ kits, mutations at codons 12 and 13 are specifically highlighted. We observed five instances of co-occurring *KRAS* mutations, specifically G12D with G12C, G13D with G12C, and a codon 12/13 mutation. Notably, we identified an exceptionally rare case involving four co-occurring *KRAS* mutations—G12D, G13D, Q61R, and A146T—in a 60-year-old male patient diagnosed with stage IVB adenocarcinoma (T4, N3, M1c).

### 4.4. PIK3CA Mutations

*PIK3CA* encodes the lipid kinase PIK3, which plays a crucial role in regulating various cellular functions such as proliferation, cell survival, degranulation, vesicular trafficking, and cell migration. Additionally, PIK3 is a key component of the PIK3/AKT signaling pathway, which is significant in the processes of oncogenesis and the progression of lung cancer [49]. *PIK3CA* mutations occur infrequently in NSCLC, with a prevalence of 1–4%. The majority of these mutations are missense mutations located in exon 9, specifically c.1624G>A (p.E542K) and c.1633G>A (p.E545K), as well as in exon 20, including c.3140A (p.H1047R), p.M1043I, and p.G1049S. These mutations affect regions that are part of the helical and kinase domains. The relationships between *PIK3CA* mutations and age, sex, smoking status, histology and lymph node metastasis have been unclear up to now [50]. *PIK3CA* mutations are correlated with both favorable and unfavorable prognoses of NSCLC patients, as they correlate with worse OS. In particular, for patients undergoing treatment with *EGFR* TKIs [48], there is evidence of poor PFS and cancer-specific survival [50], as well as a correlation with lymph node metastases [50]. Additionally, it is likely that they are not linked to primary resistance to *EGFR* TKIs in patients with lung cancer. Furthermore, the occurrence of acquired PIK3CA mutations associated with *EGFR* TKI treatment is quite uncommon [49].

In our retrospective cohort, *PIK3CA* mutations were observed in 1.82%; the frequency is consistent with previous studies [48]. Of them, eight were point mutations on exon 9 (E542K and E545K) and two were point mutations on exon 20 (H1047K). *PIK3CA* mutations were not mutually exclusive with *KRAS* mutation (G12C alone or with G12D+E542K, E545K and H1047R in three different patients), as it was for *EGFR* (one patient with H1047R *PIK3CA* + L858R *EGFR*), in opposition to previous reports [48].

### 4.5. ALK and ROS1 Rearrangements

*ALK* and *ROS1* are receptor tyrosine kinases that significantly contribute to the activation of various signaling pathways related to differentiation, proliferation, cell growth, and survival. In NSCLC, these kinases can undergo rearrangements, leading to dysregulation and inappropriate signaling through the *ALK/ROS1* kinase domain and the formation of novel fusion genes. This results in the deregulation of kinase activity and the abnormal activation of signaling pathways [51]. Approximately 2–7% of patients with NSCLC demonstrate *ALK* rearrangements, predominantly in those with adenocarcinoma histology and who are light or never smokers [12]. According to NCCN guidelines, *ALK*-positive patients are resistant to *EGFR* TKIs and should be treated with targeted agents such as alectinib, brigatinib, ceritinib, crizotinib and lorlatinib [3], although the latter has the lowest PFS and CNS activity [12]. *ROS1* rearrangements occur in 1–2.6% of NSCLC patients [52,53] and are clinically associated with never-smoking history, younger age and adenocarcinoma histological type, frequently with brain metastases [51]. Patients exhibiting ROS1 rearrangements ought to receive treatment with crizotinib, which has a response rate of 72% and a median PFS of 19.2 months [54].

From our cohort, 5.31% of the patients had *ALK* rearrangements, the frequency being in accordance with the established range. No *ROS1* rearrangements were identified in our study, which is in accordance with findings from some previous studies, identifying *ROS1* rearrangements and fusions that seldom coincide with modifications in *EGFR*, *KRAS*, *ALK* or other actionable oncogenes in NSCLC [55], although there are studies with contrasting findings (e.g., [56]) indicating rare co-mutations with *EGFR* (exon 19 deletions, exon 20 insertions, L858R) and extremely rare *BRAF*, *ALK* and *KRAS* co-mutations [55].

### 4.6. PD-L1 and HER2/NEU Expression

PD-L1 is a co-regulatory molecule that may be present on tumor cells and can suppress T-cell mediated apoptosis. While it is not the ideal choice, *PD-L1* expression currently serves as the most effective biomarker for evaluating suitability for immunotherapy [57]. In our study, 10.45% of the patients had *PD-L1* overexpression.

*HER2*, which has been validated as an emerging driver and therapeutic target in both in vitro and in vivo studies for NSCLC, encodes a member of the erbB receptor tyrosine kinase family. The occurrence of *HER2* alterations (including mutation, overexpression, and amplification) among NSCLC patients ranges from 2.4% to 38.0%, with a higher frequency observed in adenocarcinomas that exhibit well-differentiated histology. While the diagnostic significance of *HER2* alterations remains largely ambiguous, *HER2* overexpression is regarded as a negative prognostic indicator, suggesting a lower likelihood of survival [58]. Three recent studies involving 190 [33], 68 [59] and 186 [60] NSCLC patients suggest that *HER2* overexpression is not associated with the objective response to chemoradiotherapy, i.e., is not predictive of response to chemotherapy or survival (gefitinib [33], 5-fluorouracil/cisplatin/hyperfractionated RT and docetaxel/cisplatin/RT [59] and cisplatin/vinblastine, followed by RT, with or without concurrent chemotherapy with carboplatin [60]). The influence of *HER2* overexpression on sensitivity to *EGFR* TKIs, and thus RR and PFS and OS in *EGFR* mutant lung cancers, is highly debatable, with many studies for and against the positive influence on sensitivity and resistance to TKIs, giving controversial results due to small sample sizes and significant heterogeneity [58]. Patients with *HER2* overexpression should be treated with ado-trastuzumab emtansine and fam-trastuzumab deruxtecan-nxki [3].

### 4.7. Co-Occurring Genetic Alterations

The compilation of co-occurring genomic alterations in NSCLC may have a greater influence on tumor heterogeneity than the individual mutations found in oncogenic drivers. Notably, NSCLC adenocarcinomas and squamous cell carcinomas exhibit an average number of somatic mutations that surpasses that observed in numerous other cancer types [61].

In this cohort study, the majority of oncogenic driver mutations tend to occur in a mutually exclusive manner. Despite this, we detected 22 patients (3.65%) with two or more putative driver alterations. *PD-L1* alterations were most commonly seen together with *ALK* rearrangements, *KRAS* (G12V, codon 12/13), *PIK3CA* (E545K), *BRAF*, *HER2* and *EFR*. Other co-occurrences include *KRAS/PIK3CA*, *KRAS/ALK*, *KRAS/HER2*, *KRAS/BRAF*, *EGFR/PIK3CA*, and *ALK/PIK3CA*, and two patients had identical triple alterations in *KRAS/ALK/PD-L1*. All of the identified co-occurring alterations in our study are well established based on data from previous studies, for example, the co-occurrence of *PIK3CA*, *EGFR* and *KRAS* in lung adenocarcinoma [62]. *EGFR* and *KRAS* mutations are known to be mutually exclusive [48], along with mutations in other established drivers, including *ERBB2* and *BRAF*. There are also rearrangements involving *ALK*, *ROS1*, and *RET*, which predominantly do not overlap with *KRAS* mutations [61]; this was also the case in our study. The clinical significance of most of the co-occurring alterations at the time of primary diagnosis remains to be investigated, although for some co-occurring alterations it is already well established, such as, for example, the co-occurrence of *EGFR* and *PIK3CA* mutations, which is observed in 9.0–12.4% of advanced NSCLC adenocarcinomas [61]. In vitro studies indicate that the presence of both *EGFR* and *PIK3CA* mutations enhances cellular invasion and migration. Conversely, in vivo research suggests that these mutations may correlate with poorer overall survival in certain studies; however, they do not appear to influence RR or PFS when patients receive first- or second-line *EGFR* TKI therapy [63].

Unfortunately, in our study, the analysis of genetic alterations by specific histological subtypes was not relevant due to the significantly lower number of patients with squamous cell NSCLC than adenocarcinoma (4.48% vs. 92.87%) and the lower number of squamous-cell cancer patients with genetic mutation (2.82% of all patients) than with adenocarcinoma (32.84% of all patients), not considering co-occurring mutations. Research has established that mutations in *EGFR* and *KRAS*, as well as *EML4-ALK* fusions, represent the most common driver alterations in lung adenocarcinoma. These alterations occur with mutual exclusivity in approximately 35–40% of tumors. Additionally, alterations in *HER2* and *MAP2K1/MEK1* are also mutually exclusive of mutations in *PIK3CA*, *BRAF*, *EGFR*, and *KRAS*. In squamous-cell NSCLC, the most significant alterations include amplifications of *SOX2*, *PIK3CA*, *PDGFRA*, and *FGFR1*, along with mutations in *DDR2*, *AKT1*, and *NRF2*. Furthermore, mutations in *TP53*, *BRAF*, *PIK3CA* and *MET* are frequently observed in both histological subtypes [64].

## 5. Conclusions and Limitations

This study reported the frequency of gene mutations in a representative portion of lung cancer patients from North Macedonia for the first time. Reported gene alteration frequencies and patterns correspond to data reported in the existing scientific literature. Nonetheless, it is important to acknowledge several possible limitations of this study that must be taken into account:

(1) The absence of clinical follow-up data results in a lack of correlation between detected mutations, survival outcomes and treatment responses. Consequently, this study offers limited insights into the diagnostic and prognostic significance of multigene testing in the context of lung cancer diagnostics and treatment.
(2) It is possible that genetic changes occurring outside the hotspot regions addressed by the specific assays may have been missed.
(3) Tumor biopsy specimens possess an intrinsic limitation in that they fail to capture inter-metastatic tumor heterogeneity. While driver mutations are regarded as truncal events that are present across all disease sites, other co-occurring genetic alterations may have developed subsequently and could exist at locations distinct from the site where the biopsy was conducted. Liquid biopsies (such as circulating tumor DNA analysis) and advanced sequencing technologies may assist in addressing these limitations.
(4) The use of a retrospective database limits the ability to investigate other sources of potential bias.
(5) All participants in the study were Caucasian; therefore, the results obtained may not be relevant to other racial and ethnic groups.

An additional concern to address is the referral bias in the enrollment of patients who are perceived to have a higher likelihood of possessing targetable oncogenic mutations, based on clinical and demographic characteristics. This concern is evidenced by the notably higher incidence of men compared to women in the study population (70.31% vs. 29.68%) included in the study. Nonetheless, our genotyping results align with the documented prevalence of mutations in the tested oncogenes.

In conclusion, our experience with the implementation of systematic prospective genotyping for somatic alterations in genes such as *EGFR*, *BRAF*, *KRAS*, *ALK*, *ROS1*, *HER2*, *PD-L1*, and *PIK3CA* illustrates the practicality of this method within clinical workflows. The insights obtained from this approach are designed to enhance diagnostic decision-making and to inform the administration of available targeted therapies, particularly in the context of first-line chemotherapy and personalized treatment plans. As a result, the clinical feasibility of this multigene testing approach is significantly greater than that of single-gene testing. From an economic perspective, multigene testing is increasingly cost-effective in centralized laboratories or high-volume cancer centers. However, in North Macedonia, which operates within a resource-limited setting, the feasibility of multigene testing for lung cancer diagnosis and treatment is somewhat diminished compared to developed nations, primarily due to constraints related to infrastructure and reimbursement policies.

The advent of targeted therapy and immunotherapy has brought about a substantial change in the management of advanced NSCLC, demonstrating superior effectiveness when compared to chemotherapy alone in both first- and second-line treatment scenarios [18]. As a result, the next stage in the accurate diagnosis and treatment of lung cancer will focus on identifying new molecular markers. It is expected that the range of treatable oncogenic changes and prognostic biomarkers will significantly expand [65].

## Figures and Tables

**Table 1:** Overview of the patient demographics and characteristics included in the study.

Variable	*n* (%)
**gender**	
male	424 (70.32%)
female	179 (29.68%)
**age** mean ± SD	62.56 ± 9.34
<39 years	17 (2.82%)
40–49 years	41 (6.80%)
50–59 years	111 (18.40%)
60–69 years	216 (35.82%)
70–79 years	205 (34.00%)
≥80 years	13 (2.16%)
**diagnosis**	
NSCLC	598 (99.17%)
adenocarcinoma	560 (92.87%)
squamous-cell carcinoma	27 (4.48%)
large-cell carcinoma	11 (1.82%)
SCLC	5 (0.83%)

**Table 2:** Demographics of patients with gene mutations.

Gene	Number of Patients Examined (% of All Patients)	Gender		Average Age of Patients with Gene Mutations	Average Age of *wt* Patients	Diagnosis			
	Number of Patients with Gene Mutation (% of Tested)	M	F			*Adenocarcinoma*	Squamous-Cell Cancer	LCLC	SCLC
***BRAF***	432 (71.64%) 4 (0.93%)	3	1	57.75	62.87	3	1	-	-
***EGFR***	527 (87.40%) 45 (8.54%)	23	22	61.36	62.82	45 (+2 planocellular cancer)	-	-	-
***KRAS***	193 (32.00%) 50 (25.91%)	34	16	61.25	64.25	44	2 (+1 patient with adenosquamous cancer)	1	-
***PIK3CA***	142 (23.55%) 11 (7.75%)	6	5	66.91	61.86	9 (+1 patient with planocellular cancer)	1	-	-
***ALK***	411 (68.04%) 32 (7.79%)	22	10	60.94	62.81	26 (+2 planocellular cancer)	3	1	-
***PD-L1***	264 (43.71%) 63 (23.86%)	47	15	63.12	62.35	50 (+6 planocellular cancer)	4 (+1 patient with adenosquamous cancer)	1	1
***ROS-1***	117 (19.37%) 0 (0%)	0	-	-	-	-	-	-	-
***HER2/NEU***	144 (23.84%) 16 (11.11%)	12	4	63.81	62.22	9 (+1 planocellular cancer)	4	1	-

**Table 3:** Demographics of patients with concomitant gene alterations.

Patient	Diagnosis	Gender	Age	Gene 2	Gene 2	Gene 3
P1	*Adenocarcinoma*	M	71	KRAS codon 12/13	ALK	PD-L1
P2	*Carcinoma* *bronchii*	M	65	-	ALK	PD-L1
P3	*Carcinoma planocellulare*	M	64	-	ALK	PD-L1
P4	*Adenocarcinoma*	M	67	G12D, G12C KRAS	H1047R PIK3CA	-
P5	*Adenocarcinoma*	M	61	G12C KRAS	ALK	-
P6	*Adenocarcinoma*	M	66	KRAS codon 12/13	BRAF V600E	-
P7	*Adenocarcinoma*	M	49	-	ALK	PD-L1
P8	*Carcinoma bronhii*	M	68	G12C KRAS	BRAF V600E	-
P9	*Adenocarcinoma*	F	60	V600E BRAF	-	PD-L1
P10	*Adenocarcinoma*	F	60	G12C KRAS	E542K PIK3CA	-
P11	*Adenocarcinoma*	F	56	G12C KRAS	HER2	-
P12	*Adenocarcinoma*	M	59	KRAS c. s346G>T	ALK	-
P13	*Adenocarcinoma*	M	74	-	HER2	PD-L1
P14	*Adenocarcinoma*	M	37	-	EGFR	PD-L1
P15	*Adenocarcinoma*	F	72	G12V KRAS	-	PD-L1
P16	*Adenocarcinoma*	F	61	L858R EGFR	H1047R PIK3CA	-
P17	*Adenocarcinoma*	F	67	E545K PIK3CA	ALK	-
P18	*Adenocarcinoma*	M	64	-	HER2	PD-L1
P19	*Adenocarcinoma*	M	66	G12C KRAS	HER2	-
P20	*Adenocarcinoma*	M	67	G12D, G12C KRAS	E545K PIK3CA	-
P21	*Adenocarcinoma*	F	59	KRAS codon 12/13	-	PD-L1
P22	*Adenocarcinoma*	M	74	KRAS codon 12/13	ALK	PD-L1

## Data Availability

The data presented in this study are available on request from the corresponding author. The data are not publicly available due to privacy and ethical restrictions.

## Abbreviations

The following abbreviations are used in this manuscript:

*ALK*	Anaplastic lymphoma kinase
COPD	Chronic obstructive pulmonary disease
*EGFR*	Epidermal growth factor receptor
FFPE	Formalin-fixed paraffin-embedded
*HER2*	Human epidermal growth factor receptor 2
IARC	International Agency for Research on Cancer
ICI	Immune checkpoint inhibitor
*KRAS*	Kirsten rat sarcoma virus
LCLC	Large-cell lung carcinoma
LCNEC	Large-cell neuroendocrine carcinoma
NCCN	National Comprehensive Cancer Network
NOS	Lung cancer not otherwise specified
NSCLC	Non-small-cell lung cancer
OS	Overall survival
PCR	Polymerase-chain reaction
*PD-L1*	Programmed (cell) death-ligand 1
PFS	Progression-free survival
*PIK3CA*	Phosphatidylinositol-4,5-bisphosphate 3-kinase catalytic subunit alpha
*ROS-1*	Repressor of silencing 1
RR	Response rate
RT	Radiotherapy
SCLC	Small-cell lung cancer
TKI	Tyrosine kinase inhibitors
UICC	Union of International Cancer Control
WHO	World Health Organization

## References

[1] Sung H., Ferlay J., Siegel R.L., Laversanne M., Soerjomataram I., Jemal A., Bray F. (2024). Global Cancer Statistics 2022: GLOBOCAN Estimates of Incidence and Mortality Worldwide for 36 Cancers in 185 Countries. CA A Cancer J. Clin..

[2] Ferlay J., Ervik M., Lam F., Colombet M., Mery L., Piñeros M., Znaor A., Soerjomataram I., Bray F. (2020). Global Cancer Observatory: Cancer Today.

[3] Ettinger D.S., Wood D.E., Aisner D.L., Akerley W., Bauman J.R., Bharat A., Bruno D.S., Chang J.Y., Chirieac L.R., D’Amico T.A. (2022). Non-Small Cell Lung Cancer, Version 3.2022, NCCN Clinical Practice Guidelines in Oncology. J. Natl. Compr Canc. Netw..

[4] Fraumeni J.F. (1975). Respiratory Carcinogenesis: An Epidemiologic Appraisal. J. Natl. Cancer Inst..

[5] Janerich D.T., Thompson D.W., Varela L.R., Greenwald P., Chorost S., Tucci C., Zaman M., Melamed M., Kiely M., McKneally M.F. (1990). Lung cancer and exposure to tobacco smoke. N. Engl. J. Med..

[6] Driscoll T., Nelson D.I., Steenland K., Leigh J., Concha-Barrientos M., Fingerhut M., Prüss-Üstün A. (2005). The global burden of disease due to occupational carcinogens. Am. J. Ind. Med..

[7] Straif K., Benbrahim-Tallaa L., Baan R., Grosse Y., Secretan B., El Ghissassi F., Bouvard V., Guha N., Freeman C., Galichet L. (2009). A review of human carcinogens—Part C: Metals, arsenic, dusts, and fibres. Lancet Oncol..

[8] Wiesweg M., Eberhardt W.E.E., Reis H., Ting S., Savvidou N., Skiba C., Herold T., Christoph D.C., Meiler J., Worm K. (2017). High Prevalence of Concomitant Oncogene Mutations in Prospectively Identified Patients with ROS1-Positive Metastatic Lung Cancer. J. Thorac. Oncol..

[9] Travis W.D., Brambilla E., Nicholson A.G., Yatabe Y., Austin J.H.M., Beasley M.B., Chirieac L.R., Dacic S., Duhig E., Flieder D.B. (2015). The 2015 World Health Organization Classification of Lung Tumors. J. Thorac. Oncol..

[10] Howlander N., Noone A., Krapcho M., Miller D., Brest A., Yu M., Ruhl J., Tatalovic Z., Mariotto A., Lewis R. (2021). SEER Cancer Statistics Review (CSR) 1975–2018.

[11] Tsao D.-A., Chang H.-J., Lin C.-Y., Hsiung S.-K., Huang S.-E., Ho S.-Y., Chang M.-S., Chiu H.-H., Chen Y.-F., Cheng T.-L. (2010). Gene Expression Profiles for Predicting the Efficacy of the Anticancer Drug 5-Fluorouracil in Breast Cancer. DNA Cell Biol..

[12] Mithoowani H., Febbraro M. (2022). Non-Small-Cell Lung Cancer in 2022: A Review for General Practitioners in Oncology. Curr. Oncol..

[13] Keller S.M., Komaki R., Johnson D.H. (2000). A Randomized Trial of Postoperative Adjuvant Therapy in Patients with Completely Resected Stage II or IIIa Non–Small-Cell Lung Cancer. N. Engl. J. Med..

[14] Bradley J.D., Paulus R., Graham M.V., Ettinger D.S., Johnstone D.W., Pilepich M.V., Machtay M., Komaki R., Atkins J., Curran W.J. (2005). Phase II Trial of Postoperative Adjuvant Paclitaxel/Carboplatin and Thoracic Radiotherapy in Resected Stage II and IIIA Non-Small-Cell Lung Cancer Promising Long-Term Results of the Radiation Therapy Oncology Group-RTOG 9705. J. Clin. Oncol..

[15] Kong F.-M., Pan C., Eisbruch A., Haken R.K.T. (2007). Physical Models and Simpler Dosimetric Descriptors of Radiation Late Toxicity. Semin. Radiat. Oncol..

[16] Sonett J.R., Suntharalingam M., Edelman M.J., Patel A.B., Gamliel Z., Doyle A., Hausner P., Krasna M. (2004). Pulmonary Resection After Curative Intent Radiotherapy (>59 Gy) and Concurrent Chemotherapy in Non–Small-Cell Lung Cancer. Ann. Thorac. Surg..

[17] Bonanno L., Favaretto A., Rosell R. (2014). Platinum Drugs and DNA Repair Mechanisms in Lung Cancer. Anticancer Res..

[18] Leonetti A., Wever B., Mazzaschi G., Assaraf Y.G., Rolfo C., Quaini F., Tiseo M., Giovannetti E. (2019). Molecular basis and rationale for combining immune checkpoint inhibitors with chemotherapy in non-small cell lung cancer. Drug Resist. Updates.

[19] Lindeman N.I., Cagle P.T., Aisner D.L., Arcila M.E., Beasley M.B., Bernicker E.H., Colasacco C., Dacic S., Hirsch F.R., Kerr K. (2018). Updated Molecular Testing Guideline for the Selection of Lung Cancer Patients for Treatment with Targeted Tyrosine Kinase Inhibitors: Guideline From the College of American Pathologists, the International Association for the Study of Lung Cancer, and the Association for Molecular Pathology. Arch. Pathol. Lab. Med..

[20] Sobin L.H., Gospodarowicz M.K., Witterkind C. (2016). TNM Classification of Malignant Tumours.

[21] Sequist L.V., Yang J.C.-H., Yamamoto N., O’Byrne K., Hirsh V., Mok T., Geater S.L., Orlov S., Tsai C.-M., Boyer M. (2013). Phase III Study of Afatinib or Cisplatin Plus Pemetrexed in Patients with Metastatic Lung Adenocarcinoma with *EGFR* Mutations. J. Clin. Oncol..

[22] Ramalingam S.S., Vansteenkiste J., Planchard D., Cho B.C., Gray J.E., Ohe Y., Zhou C., Reungwetwattana T., Cheng Y., Chewaskulyong B. (2020). Overall Survival with Osimertinib in Untreated, EGFR-Mutated Advanced NSCLC. N. Engl. J. Med..

[23] Rosell R., Carcereny E., Gervais R., Vergnenegre A., Massuti B., Felip E., Palmero R., Garcia-Gomez R., Pallares C., Sanchez J.M. (2012). Erlotinib versus standard chemotherapy as first-line treatment for European patients with advanced EGFR mutation-positive non-small-cell lung cancer (EURTAC): A multicentre, open-label, randomised phase 3 trial. Lancet Oncol..

[24] Mitsudomi T., Morita S., Yatabe Y., Negoro S., Okamoto I., Tsurutani J., Seto T., Satouchi M., Tada H., Hirashima T. (2010). Gefitinib versus cisplatin plus docetaxel in patients with non-small-cell lung cancer harbouring mutations of the epidermal growth factor receptor (WJTOG3405): An open label, randomised phase 3 trial. Lancet Oncol..

[25] Miller V.A., Riely G.J., Zakowski M.F., Li A.R., Patel J.D., Heelan R.T., Kris M.G., Sandler A.B., Carbone D.P., Tsao A. (2008). Molecular Characteristics of Bronchioloalveolar Carcinoma and Adenocarcinoma, Bronchioloalveolar Carcinoma Subtype, Predict Response to Erlotinib. J. Clin. Oncol..

[26] Sequist L.V., Martins R.G., Spigel D., Grunberg S.M., Spira A., Jänne P.A., Joshi V.A., McCollum D., Evans T.L., Muzikansky A. (2008). First-Line Gefitinib in Patients with Advanced Non–Small-Cell Lung Cancer Harboring Somatic *EGFR* Mutations. J. Clin. Oncol..

[27] Hirsch F.R., Bunn P.A. (2009). EGFR testing in lung cancer is ready for prime time. Lancet Oncol..

[28] Paik P.K., Varghese A.M., Sima C.S., Moreira A.L., Ladanyi M., Kris M.G., Rekhtman N. (2012). Response to Erlotinib in Patients with EGFR Mutant Advanced Non-Small Cell Lung Cancers with a Squamous or Squamous-like Component. Mol. Cancer Ther..

[29] Shigematsu H., Lin L., Takahashi T., Nomura M., Suzuki M., Wistuba I.I., Fong K.M., Lee H., Toyooka S., Shimizu N. (2005). Clinical and Biological Features Associated with Epidermal Growth Factor Receptor Gene Mutations in Lung Cancers. J. Natl. Cancer Inst..

[30] Eberhard D.A., Johnson B.E., Amler L.C., Goddard A.D., Heldens S.L., Herbst R.S., Ince W.L., Jänne P.A., Januario T., Johnson D.H. (2005). Mutations in the Epidermal Growth Factor Receptor and in KRAS Are Predictive and Prognostic Indicators in Patients with Non–Small-Cell Lung Cancer Treated with Chemotherapy Alone and in Combination with Erlotinib. J. Clin. Oncol..

[31] Lee C.K., Wu Y.-L., Ding P.N., Lord S.J., Inoue A., Zhou C., Mitsudomi T., Rosell R., Pavlakis N., Links M. (2015). Impact of Specific Epidermal Growth Factor Receptor (EGFR) Mutations and Clinical Characteristics on Outcomes After Treatment with EGFR Tyrosine Kinase Inhibitors Versus Chemotherapy in EGFR -Mutant Lung Cancer: A Meta-Analysis. J. Clin. Oncol..

[32] Wu Y.-L., Zhou C., Liam C.-K., Wu G., Liu X., Zhong Z., Lu S., Cheng Y., Han B., Chen L. (2015). First-line erlotinib versus gemcitabine/cisplatin in patients with advanced EGFR mutation-positive non-small-cell lung cancer: Analyses from the phase III, randomized, open-label, ENSURE study. Ann. Oncol..

[33] Cappuzzo F., Ligorio C., Toschi L., Rossi E., Trisolini R., Paioli D., Magrini E., Finocchiaro G., Bartolini S., Cancellieri A. (2007). EGFR and HER2 Gene Copy Number and Response to First-Line Chemotherapy in Patients with Advanced Non-small Cell Lung Cancer (NSCLC). J. Thorac. Oncol..

[34] Zhou C., Ramalingam S.S., Kim T.M., Kim S.-W., Yang J.C.-H., Riely G.J., Mekhail T., Nguyen D., Garcia-Campelo M.R., Felip E. (2021). Treatment Outcomes and Safety of Mobocertinib in Platinum-Pretreated Patients with EGFR Exon 20 Insertion–Positive Metastatic Non–Small Cell Lung Cancer. JAMA Oncol..

[35] Yasuda H., Park E., Yun C.-H., Sng N.J., Lucena-Araujo A.R., Yeo W.-L., Huberman M.S., Cohen D.W., Nakayama S., Ishioka K. (2013). Structural, biochemical and clinical characterization of epidermal growth factor receptor (EGFR) exon 20 insertion mutations in lung cancer. Sci. Transl. Med..

[36] Riess J.W., Gandara D.R., Frampton G.M., Madison R., Peled N., Bufill J.A., Dy G., Ou S.-H.I., Stephens P.J., McPherson J. (2018). Diverse EGFR Exon 20 Insertions and Co-Occurring Molecular Alterations Identified by Comprehensive Genomic Profiling of Non-Small Cell Lung Cancer. J. Thorac. Oncol..

[37] Riely G.J., Neal J.W., Camidge D.R., Spira A.I., Piotrowska Z., Costa D.B., Tsao A.S., Patel J.D., Gadgeel S.M., Bazhenova L. (2021). Activity and Safety of Mobocertinib (TAK-788) in Previously Treated Non–Small Cell Lung Cancer with EGFR Exon 20 Insertion Mutations From a Phase 1/2 Trial. Cancer Discov..

[38] Park K., Haura E.B., Leighl N.B., Mitchell P., Shu C.A., Girard N., Viteri S., Han J.-Y., Kim S.-W., Lee C.K. (2021). Amivantamab in EGFR Exon 20 Insertion–Mutated Non–Small-Cell Lung Cancer Progressing on Platinum Chemotherapy: Initial Results From the CHRYSALIS Phase I Study. J. Clin. Oncol..

[39] Udagawa H., Matsumoto S., Ohe Y., Satouchi M., Furuya N., Kim Y.H., Seto T., Soejima K., Hayakawa D., Kato T. (2019). OA07.03 Clinical Outcome of Non-Small Cell Lung Cancer with EGFR/HER2 Exon 20 Insertions Identified in the LC-SCRUM-Japan. J. Thorac. Oncol..

[40] Ou S.-H.I., Lin H., Hong J.-L. (2021). Real-world response and outcomes in NSCLC patients with EGFR exon 20 insertion mutations. J. Clin. Oncol..

[41] Vasconcelos P.E.N.S., Gergis C., Viray H., Varkaris A., Fujii M., Rangachari D., VanderLaan P.A., Kobayashi I.S., Kobayashi S.S., Costa D.B. (2020). EGFR-A763_Y764insFQEA Is a Unique Exon 20 Insertion Mutation That Displays Sensitivity to Approved and In-Development Lung Cancer EGFR Tyrosine Kinase Inhibitors. JTO Clin. Res. Rep..

[42] Yasuda H., Kobayashi S., Costa D.B. (2012). EGFR exon 20 insertion mutations in non-small-cell lung cancer: Preclinical data and clinical implications. Lancet Oncol..

[43] Chelabi S., Mignard X., Leroy K., Monnet I., Brosseau S., Theou-Anton N., Massiani M.-A., Friard S., Duchemann B., Fabre E. (2021). EGFR Exon 20 Insertion in Metastatic Non-Small-Cell Lung Cancer: Survival and Clinical Efficacy of EGFR Tyrosine-Kinase Inhibitor and Chemotherapy. Cancers.

[44] Byeon S., Kim Y., Lim S.W., Cho J.H., Park S., Lee J., Sun J.-M., Choi Y.-L., Lee S.-H., Ahn J.S. (2019). Clinical Outcomes of EGFR Exon 20 Insertion Mutations in Advanced Non-small Cell Lung Cancer in Korea. Cancer Res. Treat. Off. J. Korean Cancer Assoc..

[45] Perrone F., Mazzaschi G., Minari R., Verzè M., Azzoni C., Bottarelli L., Nizzoli R., Pluchino M., Altimari A., Gruppioni E. (2022). Multicenter Observational Study on Metastatic Non-Small Cell Lung Cancer Harboring BRAF Mutations: Focus on Clinical Characteristics and Treatment Outcome of V600E and Non-V600E Subgroups. Cancers.

[46] Slebos R.J.C., Hruban R.H., Dalesio O., Mooi W.J., Offerhaus G.J.A., Rodenhuis S. (1991). Relationship Between K-ras Oncogene Activation and Smoking in Adenocarcinoma of the Human Lung. J. Natl. Cancer Inst..

[47] Shen M., Qi R., Ren J., Lv D., Yang H. (2022). Characterization with KRAS Mutant Is a Critical Determinant in Immunotherapy and Other Multiple Therapies for Non-Small Cell Lung Cancer. Front. Oncol..

[48] Ludovini V. (2011). Phosphoinositide-3-Kinase Catalytic Alpha and KRAS Mutations are Important Predictors of Resistance to Therapy with Epidermal Growth Factor Receptor Tyrosine Kinase Inhibitors in Patients with Advanced Non-small Cell Lung Cancer. J. Thorac. Oncol..

[49] Wu S.-G., Chang Y.-L., Yu C.-J., Yang P.-C., Shih J.-Y. (2016). The Role of PIK3CA Mutations among Lung Adenocarcinoma Patients with Primary and Acquired Resistance to EGFR Tyrosine Kinase Inhibition. Sci. Rep..

[50] Wang Y., Wang Y., Li J., Li J., Che G. (2020). Clinical Significance of PIK3CA Gene in Non-Small-Cell Lung Cancer: A Systematic Review and Meta-Analysis. BioMed Res. Int..

[51] Gendarme S., Bylicki O., Chouaid C., Guisier F. (2022). ROS-1 Fusions in Non-Small-Cell Lung Cancer: Evidence to Date. Curr. Oncol..

[52] Dugay F., Llamas-Gutierrez F., Gournay M., Medane S., Mazet F., Chiforeanu D.C., Becker E., Lamy R., Léna H., Rioux-Leclercq N. (2017). Clinicopathological characteristics of ROS1- and RET-rearranged NSCLC in caucasian patients: Data from a cohort of 713 non-squamous NSCLC lacking KRAS/EGFR/HER2/BRAF/PIK3CA/ALK alterations. Oncotarget.

[53] Kim H.R., Lim S.M., Kim H.J., Hwang S.K., Park J.K., Shin E., Bae M.K., Ou S.-H.I., Wang J., Jewell S.S. (2013). The frequency and impact of ROS1 rearrangement on clinical outcomes in never smokers with lung adenocarcinoma. Ann. Oncol..

[54] Shaw A.T., Ou S.-H.I., Bang Y.-J., Camidge D.R., Solomon B.J., Salgia R., Riely G.J., Varella-Garcia M., Shapiro G.I., Costa D.B. (2014). Crizotinib in ROS1 -Rearranged Non–Small-Cell Lung Cancer. N. Engl. J. Med..

[55] Lin J.J., Ritterhouse L.L., Ali S.M., Bailey M., Schrock A.B., Gainor J.F., Ferris L.A., Mino-Kenudson M., Miller V.A., Iafrate A.J. (2017). ROS1 Fusions Rarely Overlap with Other Oncogenic Drivers in Non–Small Cell Lung Cancer. J. Thorac. Oncol..

[56] Lambros L., Guibourg B., Uguen A. (2018). ROS1-rearranged Non–Small Cell Lung Cancers with Concomitant Oncogenic Driver Alterations: About Some Rare Therapeutic Dilemmas. Clin. Lung Cancer.

[57] Kerr K.M., Hirsch F.R. (2016). Programmed Death Ligand-1 Immunohistochemistry: Friend or Foe?. Arch. Pathol. Lab. Med..

[58] Zhao J., Xia Y. (2020). Targeting HER2 Alterations in Non–Small-Cell Lung Cancer: A Comprehensive Review. JCO Precis. Oncol..

[59] Kuyama S., Hotta K., Tabata M., Segawa Y., Fujiwara Y., Takigawa N., Kiura K., Ueoka H., Eguchi K., Tanimoto M. (2008). Impact of HER2 Gene and Protein Status on the Treatment Outcome of Cisplatin-Based Chemoradiotherapy for Locally Advanced Non-small Cell Lung Cancer. J. Thorac. Oncol..

[60] Graziano S.L., Tatum A., Herndon J.E., Box J., Memoli V., Green M.R., Kern J.A. (2001). Use of neuroendocrine markers, p53, and HER2 to predict response to chemotherapy in patients with stage III non-small cell lung cancer: A Cancer and Leukemia Group B study. Lung Cancer.

[61] Skoulidis F., Heymach J.V. (2019). Co-occurring genomic alterations in non-small cell lung cancer biology and therapy. Nat. Rev. Cancer.

[62] Sholl L.M., Aisner D.L., Varella-Garcia M., Berry L.D., Dias-Santagata D., Wistuba I.I., Chen H., Fujimoto J., Kugler K., Franklin W.A. (2015). Multi-institutional oncogenic driver mutation analysis in lung adenocarcinoma: The Lung Cancer Mutation Consortium experience. J. Thorac. Oncol..

[63] Eng J., Woo K.M., Sima C.S., Plodkowski A., Hellmann M.D., Chaft J., Kris M.G., Arcila M.E., Ladanyi M., Drilon A. (2015). Impact of concurrent PIK3CA mutations on response to EGFR tyrosine kinase inhibition in EGFR-mutant lung cancers and on prognosis in oncogene-driven lung adenocarcinomas. J. Thorac. Oncol..

[64] Pikor L.A., Ramnarine V.R., Lam S., Lam W.L. (2013). Genetic alterations defining NSCLC subtypes and their therapeutic implications. Lung Cancer.

[65] Reck M., Rabe K.F. (2017). Precision Diagnosis and Treatment for Advanced Non–Small-Cell Lung Cancer. N. Engl. J. Med..

